# Belief in a Just World Lowers Perceived Intention of Corruption: The Mediating Role of Perceived Punishment

**DOI:** 10.1371/journal.pone.0097075

**Published:** 2014-05-16

**Authors:** Bao-yu Bai, Xiao-xiao Liu, Yu Kou

**Affiliations:** 1 Institute of Developmental Psychology, Beijing Normal University, Beijing, P. R. China; 2 Nanyang Business School, Nanyang Technological University, Singapore, Singapore; George Mason University/Krasnow Institute for Advanced Study, United States of America

## Abstract

Corruption can be unfair and detrimental to societies; however, little is known regarding how individuals perceive corruption. We aim to understand how psychological factors, such as lay belief of the world, influence perceived intention of corruptive behavior. As corruption undermines justice, we hypothesize that belief in a just world to others (BJW-others) reduces perceived intention of corruptive behaviors. We conducted two correlational studies and one experimental study in China. Using hypothetical scenarios, perception toward bribery taking and nepotistic practices were assessed. In Study 1 and Study 2, we consistently found that BJW-others negatively predicted perceived intention of corruption, and this pattern was mediated by perceived likelihood of punishment. We further replicate this result in Study 3 by priming BJW-others, demonstrating its causal effect. The results indicate that BJW as one lay belief can be important in influencing people’s attitudes toward corruption. Implications for future research and anti-corruption policies are also discussed.

## Introduction

Corruption is considered rampant throughout the world [1] and is typically defined as one’s misuse of entrusted power for his or her personal gain [2], [3]. Corruption undermines justice in a society [1], [4], [5], endangers democratic and moral values, and slows economic advancement [6]. Previous research has focused on understanding the occurrence of corruption. Factors at the country level [7]–[10] and individual level have been identified as determinants of corruption [11]–[13]. In present study, we focus on how people perceive corruption.

The importance of understanding the perception of corruption is at least twofold. Firstly, when people perceive corruption as prevalent, they may justify it as a means to achieve goals, and thus become more willing to get involved in corruption [14]–[16]. Research on perceived prevalence of corruption has identified various factors shaping this type of corruption perception [17], such as religion, economic development, democratic institutions, cultural values at the macro level [1], [18], and demographic variables at the micro level, such as age, education, residential and occupational status [18], [19].

Secondly, when people perceive that individuals in power intend to participate in corruption, they may become more willing to get involved in corruption [19]. In the current research, we define corruption perception as one’s perceived intention toward people in power in exchange for favorable treatment and personal benefits. Such treatment and benefits include financial capital, such as bribery, or relational capital, such as nepotistic practices [1], [3]. As perceived intention of corruption may influence people’s own intention to initiate corruption, we aim to examine the antecedents and mechanism of determining this type of corruption perception.

### Belief in a Just World and Corruption Perception

We propose that individuals’ lay belief of the world could shape corruption perception. It appears that corruption often leads to unfair decisions and resource distributions [9], [20]. In this regard, one’s *belief in a just world* (BJW) could be an important predictor of corruption perception. With the application of just-world theory [21], individuals hold a set of beliefs that they live in a just world and obtain what they deserve. Despite the negative consequences of BJW, which may lead to derogation of disadvantaged groups [22], BJW can be functional to regulate ones’ own behaviors. Consistent with this premise, as compared to non-believers in BJW, believers in BJW tend to achieve their goals by just means [21], [23], [24], have a high level of confidence that they will be treated fairly by others [25], [26], and have low levels of rule-breaking behavior [26] and delinquent intentions [27]. Taken together, we speculate that individuals who hold BJW tend to perceive less intention of committing corruptive behavior compared to those do not believe in BJW.

### Perceived Likelihood of Punishment as Mediator

We also anticipate that a higher BJW leads to a higher perceived likelihood of punishment of corruption because BJW leads to punitive attitudes in criminal justice [22], [28]. Research on corruption in laboratory settings [29] and real life situations [30] has found that punishment reduced corruption levels, thus it is also plausible that perceived likelihood of punishment would mediate the effect of BJW on corruption perception.

It is noteworthy that BJW is typically divided into BJW-others (belief in a just world to others) and BJW-self (belief in a just world to oneself) [31]. We expect the proposed effects of BJW on perceived punishment and corruptive behavior limit BJW-others rather than BJW-self. The rationale is that BJW-self is related to intra-individual outcomes, such as mental health and subjective well-being [31]–[33], whereas BJW-others is related to interpersonal level phenomena, such as higher levels of punitiveness toward offenders [22] or harsh attitudes toward disadvantaged groups [34], [35]. As corruption involves interpersonal dynamics and its unfair outcomes can extend to others beyond oneself, we specify BJW-others but not BJW-self as the antecedent of corruption perception.

### Overview of the Current Studies

In the present study, we hypothesize that BJW-others (but not BJW-self) would negatively predict perceived intention of corruption (Hypothesis 1). We hypothesize that this relationship would be mediated by perceived likelihood of punishment for corruptive behavior (Hypothesis 2). To test these hypotheses, we used bribery taking and nepotistic practice in Study 1 and 2, respectively, as two forms of corruption in hypothetical scenarios in surveys. In Study 3, we manipulated BJW-others with a priming paradigm and established the causal link between BJW-others, perceived likelihood of punishment, and perceived intention of corruption.

## Study 1

In this study, we aim to establish the relationship between individuals’ BJW-others and perceived intention of corruption, and the mediating role of perceived likelihood of punishment in BJW-others–corruption perception relationship.

### Ethics Statement

This research (studies 1–3) was approved by the local ethical committee of Beijing Normal University. All participants were required to read and approve the informed consent before answering the survey.

### Participants

We recruited 86 participants (54.65% male and 44.19% female, one did not indicate gender), aged 22 to 56 (*M* = 30.52, *SD* = 5.69) via Sojump, an online platform similar to Mechanical Turk, which is used to launch nationwide e-surveys in China. Occupational information was as follows: 39.1% of the participants were workers, 13.8% were college students, 24% were college teachers, 11.5% were public servants, and 11.6% Others.

### Procedure and Measures

Participants completed an eight-item scale of BJW-self (*M* = 3.86, *SD* = .80, *α* = .89) and a seven-item scale of BJW-others (*M* = 3.37, *SD* = .89, *α* = .84) in a 6-point Likert scale borrowed from Lipkus et al. (1996). A higher score indicated one’s stronger endorsement that the world was just. Subsequently, we presented three hypothetical scenarios portraying one person (favor requester) to ask someone in power (favor receiver) to help in achieving an illegal goal by offering a payment. These three scenarios were pertaining to winning in bidding, to avoiding punishments from a severe traffic regulation violation, and to obtaining an approval of academic funding. The scenarios were generated from a panel discussion among the authors. A sample scenario of winning in bidding reads:

Wu Yong-Jun is a director who is in charge of bidding. Company *X* is in a disadvantaged position compared with other bidders. To secure the bidding, the CEO of Company *X* asked Wu to help his company and promised to bribe Wu privately if his company wins the bid. Wu consciously knows that helping him to win the bid by taking the bribe is an illegal act.

After each scenario, perceived intention of corruption was measured by “please estimate the likelihood of favor receiver would offer the help” in a 9-point Likert scale ranging from 1 (*definitely will not help*) to 9 (*definitely will help*). Perceived likelihood of punishment was measured by “please estimate the likelihood of punishment if the favor receiver help in the request” in a 9-point Likert scale ranging from 1 (*definitely won’t be punished*) and 9 (*definitely will be punished*). We averaged the scores across the three scenarios as an indicator of perceived intention of corruption (*α* = .79) and perceived likelihood of punishment (*α* = .80).

Demographic information including gender, age, education, and income was collected at the end.

### Results

Table 1 reports the descriptive statistics and correlations among the variables. Previous research suggested that gender, age, education, and income level are related to corruption perception [18], [19], thus we controlled these variables in the following analysis.

**Table 1:** Descriptive statistics and correlations among the key variables in Study 1.

Variable	*M*	*SD*	Correlation						
			1	2	3	4	5	6	7
1. Gender	0.44	0.50	–						
2. Age (years)	30.52	5.69	−.13	–					
3. Education (1 to 6)	4.21	1.01	.11	−.09	–				
4. Income (Yuan, RMB)	3964.84	2255.58	−.00	.32**	−.22*	–			
5. BJW-self	3.86	0.80	.12	−.01	.31**	−.05	–		
6. BJW-others	3.37	0.89	.14	.11	.24*	.02	.75***	–	
7. Perceived likelihood of punishment	4.32	1.96	.10	.31**	−.06	.01	.15	.32**	–
8. Perceived intention of corruption	6.09	1.88	−.11	−.15	−.02	.06	−.33**	−.45***	−.64***

*Note*: Gender was dummy-coded as 0 for male and 1 for female.

*p<.05.

**p<.01.

***p<.001.

We conducted a series of hierarchical regression analyses to determine whether BJW-others predicted corruption perception while controlling for BJW-self and demographic variables. As shown in Table 2 (see Model 1 and Model 2), we first entered control variables and then the mean-centered scores of BJW-self and BJW-others as predictors. We found that adding BJW-self and BJW-others contributed to the explanation of variance in corruption perception over and above control variables. Further, BJW-others (*β* = −.47, *p*<.01), but not BJW-self (*β* = −.01, *p*>.1), predicted corruption perception. Hypothesis 1 was supported, suggesting that a higher level of endorsement of BJW-others led to a lower perceived intention that the favor receiver would take the bribe.

**Table 2:** Results of hierarchical regression equations testing the role of BJW-others on perceived intention of corruption and mediation model in Study 1.

	Model 1		Model 2		Model 3	
Variable	*β*	*t*	*β*	*t*	*β*	*t*
Gender	−.07	−.58	−.00	−.01	.03	.36
Age	−.19	−1.58	−.13	−1.20	.03	.33
Education	−.01	−.04	.11	1.01	.04	.44
Income	.11	.92	.14	1.21	.07	.70
BJW-self			−.01	−.03	−.07	−. 55
BJW-others			−.47	−2.89**	−.24	−1.70
Perceived likelihood of punishment					−.56	−5.75***
Adjusted *R* ^2^	.01		.18		.43	
*F* change	.76		9.30***		33.02***	

*Note*: Gender was dummy-coded as 0 for male and 1 for female.

**p<.01.

***p<.001.

We examined Hypothesis 2 using a regression analysis, controlling for BJW-self and demographic variables [36]. The results showed that BJW-others was negatively associated with perceived intention of corruption (*β* = −.47, *p*<.01, *f* ^2^ = .12) (see Model 2 in Table 2), and predicted higher perceived likelihood of punishment (*β* = .41, *p*<.05, *f* ^2^ = .09). When perceived likelihood of punishment was included as a predictor in the regression analysis, it emerged as a significant suppressor of perceived intention of corruption (*β* = −.56, *p*<.001, *f* ^2^ = .47), whereas the effect of BJW-others on perceived intention of corruption became non-significant (*β* = −.24, *p* = . 09) (see Model 3 in Table 2). This mediation effect was further supported by bootstrapping method (i.e., indirect effect = .40, *SE* = .19, 95% confidence interval (CI) = [−.80, −.07]; 5,000 bootstrap samples) [37]. When not including control variables, perceived likelihood of punishment partially mediated the effect of BJW-others on perceived intention of corruption (Sobel test: *z* = 2.82, *p*<.01). Hypothesis 2 was supported.

### Discussion

We found supporting evidence that BJW-others lowered the perceived intention of corruption, and this effect was mediated by perceived likelihood of punishment. As corruption comes in many forms, we aim to replicate this finding using another corruption situation. We used nepotism, another widespread form of corruption, as the target of corruption perception. We used one’s classmate and brother in the scenario, as these personal ties are considered as nepotistic and evaluated negatively in hiring practices [38].

## Study 2

We aim to replicate the findings in Study 1 using a different context and sample.

### Participants

We recruited 90 undergraduate students (40.00% male, 57.78% female, two missing data; Mean of age = 21.34, *SD* = 1.13) from a university in north China. Participants joined the study voluntarily and were given a gel pen as a gift upon the completion of the survey.

### Design and Procedure

Participants were randomly assigned to one of the two conditions (brother or classmate) in a between-subject design. We used the same scenarios as in Study 1 with an exception that instead of offering a bribe, the favor requester was either the brother or a classmate of an official. We averaged the scores across the three scenarios as an indicator of perceived intention of corruption (*α* = .77) and perceived likelihood of punishment (*α* = .76).

### Results and Discussion

**Figure 1:**
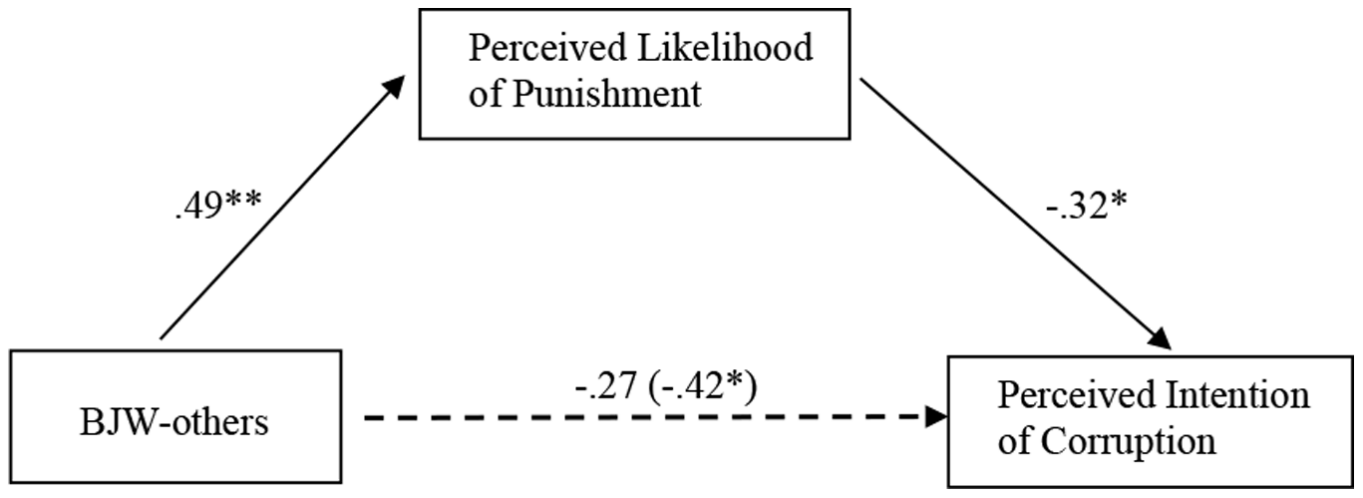
Hypothesized model linking BJW-others and corruption perception in brother condition as mediated by perceived likelihood of punishment in Study 2. *Note*: Solid lines indicate paths significant at *p*<.05, whereas the dotted line indicates a non-significant path at *p*>.05 once perceived likelihood of punishment was added into the model. Parameter estimates are standardized regression coefficients (*β*). *p<.05. **p<.01.

**Figure 2:**
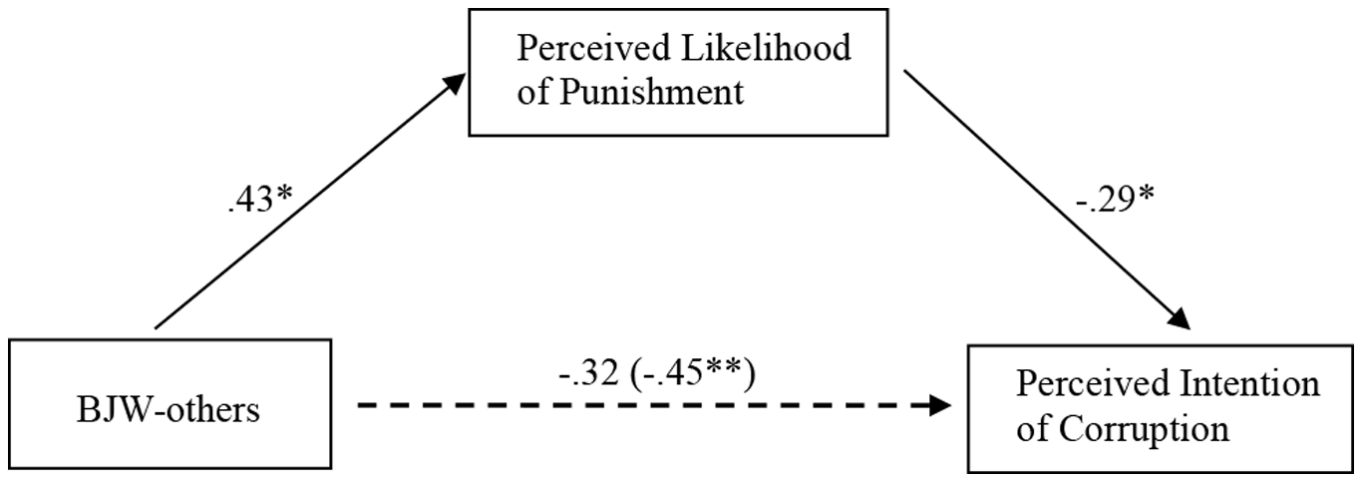
Hypothesized mediational model of perceived likelihood of punishment on BJW-others and corruption perception in classmate condition of Study 2. *Note*: *p<.05. **p<.01.

We conducted two separate mediational analyses for brother and classmate conditions with control variables. As shown in Figure 1 (brother condition) and Figure 2 (classmate condition), BJW-others was negatively associated with corruption perception (Brother condition: *β* = −.42, *p*<.05, *f* ^2^ = .17; Classmate condition: *β* = −.45, *p*<.01, *f* ^2^ = .19), and predicted higher level of perceived likelihood of punishment (Brother condition: *β* = .49, *p*<.01, *f* ^2^ = .21; Classmate condition: *β* = .43, *p*<.05, *f* ^2^ = .16). When perceived likelihood of punishment was included as a mediator, it emerged as a significant and negative predictor of corruption perception (Brother condition: *β* = −.32, *p*<.05, *f* ^2^ = .12; Classmate condition: *β* = −.29, *p*<.05, *f* ^2^ = .10), and the effect of BJW-others on corruption perception became non-significant (Brother condition: *β* = −.27, *p*>.1; Classmate condition: *β* = −.32, *p*>.05). This full mediation effect was further supported by the bootstrapping method (Brother condition: indirect effect = .55, *SE* = .37, 95% confidence interval (CI) = [−1.45, −.02]; Classmate condition: indirect effect = .42, *SE* = .23, 95% confidence interval (CI) = [−.93, −.02]; 5,000 bootstrap samples). When excluding control variables, perceived likelihood of punishment remained a significant mediator in both the brother condition (Sobel test: *z* = 2.03, *p*<.05) and the classmate condition (Sobel test: *z* = 1.91, *p*<.05). Again, the findings illustrate that perceived likelihood of punishment mediated the effect of BJW-others on corruption perception. Hypotheses 1 and 2 were again supported.

## Study 3

Hypotheses 1 and 2 were supported using correlational studies so far. In Study 3, we hope to examine the causal relationship between BJW-others and corruption perception. We attempt to manipulate BJW-others by asking participants to recall and write down just or unjust incidents or experiences. Theoretically, BJW-others is one kind of declarative knowledge. Thus, the principles of knowledge activation would apply [39]. Although there are chronic differences in people’s endorsement of BJW-others [40], this belief can be activated by experimental manipulation [41], [42], as the temporary accessibility of BJW can be heightened or dampened after recalling experiences supporting or against this belief. Therefore, recalling just versus unjust experiences would temporarily shape endorsement of BJW.

To examine the validity of this manipulation, we conducted a pilot study among 117 undergraduate students in China. In this study, we asked participants to “recall two incidents or experiences you feel just (unjust), and write them down as clearly as possible in ten minutes”. We measured BJW-others after the task as a manipulation check. As expected, BJW-others was indeed significantly higher after just world priming (*M* = 3.82, *SD* = .83) than after unjust world priming (*M* = 3.44, *SD* = .74), *F* (1, 115) = 6.60, *p*<.05, *η* ^2^ = .05. We used this manipulation in Study 3.

### Participants and Design

We recruited another 101 undergraduate students (54.46% male, 44.55% female, one did not indicate his or her gender; Mean of age = 21.29, *SD* = 1.35) in China. Participants were randomly assigned to three conditions using a between-subject design, namely high BJW group (just world priming), low BJW group (unjust world priming), and control group.

### Procedure and Measures

We asked participants to recall and write just and unjust incidents or experiences in two experimental groups. Participants from the control group were asked to recall and write a typical day at school. Then, all participants read the same nepotism scenarios as in Study 2 with only the brother as favor requester this time.

### Results

**Figure 3:**
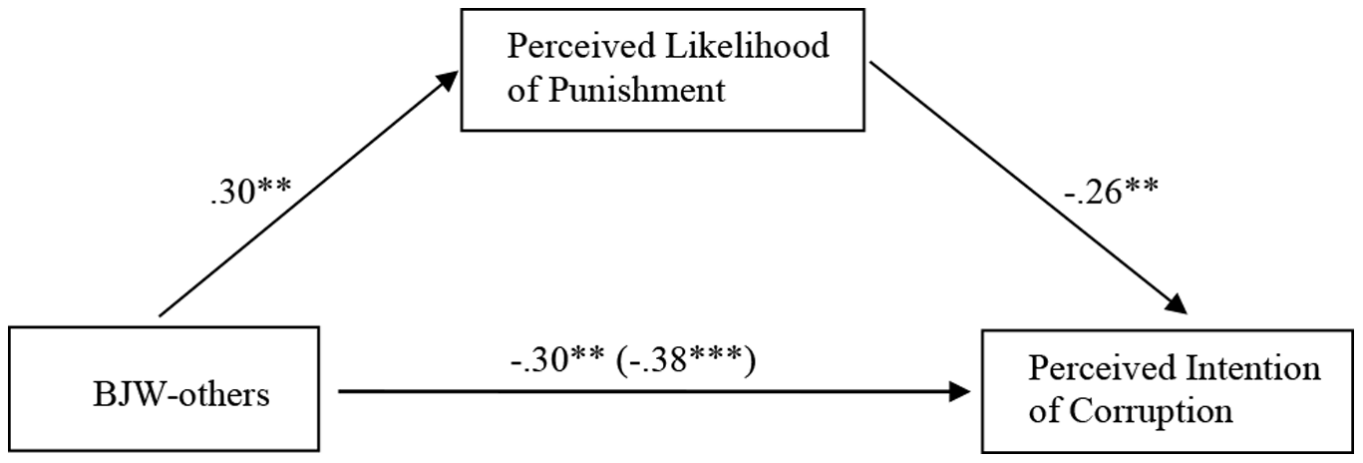
Hypothesized mediational model of perceived likelihood of punishment on BJW-others and corruption perception in Study 3. *Note*: **p<.01. ***p<.001.

We ran a one-way ANCOVA to test Hypothesis 1, while controlling for gender and age. As expected, participants from the low BJW group showed significantly higher perceived intention of corruption (*M* = 7.20, *SD* = 1.62) than the control group (*M* = 6.08, *SD* = 1.80), *F* (1, 95) = 7.66, *p*<.01, and the high BJW group (*M* = 5.39, *SD* = 1.95), *F* (1, 95) = 16.53, *p*<.001, *η* ^2^ = .16. For perceived likelihood of punishment, participants from the low BJW group (*M* = 3.72, *SD* = 1.56) perceived significantly lower likelihood of punishment than the control group (*M* = 4.94, *SD* = 2.03), *F* (1, 95) = 12.73, *p*<.01, and the high BJW group (*M* = 5.13, *SD* = 2.01), *F* (1, 95) = 10.17, *p*<.01, *η* ^2^ = .14. Moreover, this difference in perceived likelihood of punishment partially mediated the effect (*β* = −.26, *p*<.01) of priming BJW-others on perceived intention of corruption. As shown in Figure 3, although the effect of priming on perceived intention of corruption remained significant (*β* = −.30, *p*<.01), its size dropped significantly according to Sobel Test, *z* = −2.04, *p*<.05. This partial mediation effect was further supported by the bootstrapping method (Indirect effect = .18, *SE* = .10, 95% confidence interval (CI) = [−.41, −.02]; 5,000 bootstrap samples). Including control variables or not did not change this pattern. Hypothesis 2 was partially supported.

## General Discussion

As corruption undermines social justice, we aim to investigate how and why belief in a just world (BJW) influences the perception of others’ intention of committing corruption. Across three studies using online samples and undergraduate samples in China, we consistently found that BJW-others led to lower perceived corruption intention, and this effect can be at least partially mediated by a higher perceived likelihood of punishment for corruptive behavior.

These findings have three major contributions. First, we extend previous research on antecedents of corruption perception from demographic variables and moral concerns to a lay belief in a just world [18], [19], [43]. It suggests that beyond trait variables, construction of the world colors the perception of relating social phenomena. Future studies can examine the effect of other kinds of lay beliefs on corruption perception.

Second, our findings provide implications for anti-corruption policies. As lay beliefs can be observed, learned, and transmitted via texts, institutions, and discourses [44], emphasizing the importance of maintaining justice can be effective in reducing perceived corruption intention, and may eventually decrease personal intention to involve in corruption. In Study 3, we demonstrated the priming effect of BJW-others indeed lowered corruption perception, indicating the malleability of BJW-others and its effect on corruption perception. Therefore, we speculate that an emphasis on social justice may enhance BJW-others, which leads individuals not only to perceive a higher likelihood of punishment for corruption, but also a lower perception of corruption intention of others. This effect can be further examined in other unethical behaviors.

Third, the suppression effect of BJW-others on corruption perception contributes to the literature on BJW. In addition to the downside of BJW-others, such as victim blaming and derogation toward disadvantaged groups [34], [45], we reveal the bright side of this lay belief on corruption perception. Our findings imply that BJW-others can lower perceived intention of corruption, which may lead to lower intention and actual corruptive behavior. Future research may further explore the functional aspects of BJW-others on other negative social phenomena regarding violation of justice.

One limitation of the current research is that perceived likelihood of punishment sometimes partially mediated rather than fully mediated the effect of BJW-others on perceived intention of corruption. As perceived punishment is based on exogenous sanction, future research may examine the role of endogenous constructs such as moral principles or moral traits. Evidence has shown that BJW moderates the effect of cognitive moral development on ethical decision-making [46]. It is possible that BJW influences the activation of ethical scripts, which further influence judgment and decision making regarding corrupt behaviors. We call for future investigation on other potential mechanisms.

Another limitation is that we used self-report measures of all variables. The major concern of using self-report measures is common-method bias, which may exert a systematic effect (e.g., acquiescence or leniency effects) on the observed correlation between the measures [47]. Thus, the observed correlations between the measures could be inflated because of common-method bias. Meanwhile, we still use self-report measures for the following reasons. First, as corruption is always clandestine, to measure corruption is always a challenge. Second, we are interested in perceived others’ corruption intention, thus it is very difficult to come up with precise objective measures. Indeed, previous literature on corruption intention also employed self-report measures [8], [48]. Balancing the strengths and weaknesses of using self-report measures, we consider self-report measures as the best candidate of measurements so far. We are open for suggestions on resolving issues with measurements in understanding corruption intention in future studies.
